# Effects of Fisetin Treatment on Cellular Senescence of Various Tissues and Organs of Old Sheep

**DOI:** 10.3390/antiox12081646

**Published:** 2023-08-21

**Authors:** Charles A. Huard, Xueqin Gao, Maria E. Dey Hazra, Rony-Orijit Dey Hazra, Kimberly Lebsock, Jeremiah T. Easley, Peter J. Millett, Johnny Huard

**Affiliations:** 1 Linda and Mitch Hart Center for Regenerative and Personalized Medicine, Steadman Philippon Research Institute, Vail, CO 81657, USA or charleshuard@tamu.edu (C.A.H.); rony@deyhazra.de (R.-O.D.H.); drmillett@thesteadmanclinic.com (P.J.M.); 2 The Steadman Clinic, Vail, CO 81657, USA; 3 Department for Shoulder and Elbow Surgery, Center for Musculoskeletal Surgery, Charite-University Medicine Berlin, Freie Universität Berlin, Humboldt-Universität zu Berlin, Berlin Institute of Health, 14195 Berlin, Germany; 4 Preclinical Surgical Research Laboratory, Department of Clinical Sciences, Colorado State University, Fort Collins, CO 80523, USA; kimberly.menges@colostate.edu (K.L.); jeremiah.easley@colostate.edu (J.T.E.)

**Keywords:** fisetin, cellular senescence, sheep, β-galactosidase, cerebral brain cortex, cerebellum, hippocampus, neurons, astrocytes, microglia, lung, liver, spleen

## Abstract

Fisetin has been shown to be beneficial for brain injury and age-related brain disease via different mechanisms. The purpose of this study was to determine the presence of senescent cells and the effects of fisetin on cellular senescence in the brain and other vital organs in old sheep, a more translational model. Female sheep 6–7 years old (N = 6) were treated with 100 mg/kg fisetin or vehicle alone on two consecutive days a week for 8 weeks. All vital organs were harvested at the time of sacrifice. Histology, immunofluorescence staining, and RT-Q-PCR were performed on different regions of brain tissues and other organs. Our results indicated that fisetin treatment at the current regimen did not affect the general morphology of the brain. The presence of senescent cells in both the cerebral brain cortex and cerebellum and non-Cornu Ammonis (CA) area of the hippocampus was detected by senescent-associated β-galactosidase (SA-β-Gal) staining and GL13 (lipofuscin) staining. The senescent cells detected were mainly neurons in both gray and white matter of either the cerebral brain cortex, cerebellum, or non-CA area of the hippocampus. Very few senescent cells were detected in the neurons of the CA1-4 area of the hippocampus, as revealed by GL13 staining and GLB1 colocalization with NEUN. Fisetin treatment significantly decreased the number of SA-β-Gal^+^ cells in brain cortex white matter and GL13^+^ cells in the non-CA area of the hippocampus, and showed a decreasing trend of SA-β-Gal^+^ cells in the gray matter of both the cerebral brain cortex and cerebellum. Furthermore, fisetin treatment significantly decreased P16^+^ and GLB1^+^ cells in neuronal nuclear protein (NEUN)^+^ neurons, glial fibrillary acidic protein (GFAP)^+^ astrocytes, and ionized calcium binding adaptor molecule 1 (IBA1)^+^ microglia cells in both gray and white matter of cerebral brain cortex. Fisetin treatment significantly decreased GLB1^+^ cells in microglia cells, astrocytes, and NEUN^+^ neurons in the non-CA area of the hippocampus. Fisetin treatment significantly decreased plasma S100B. At the mRNA level, fisetin significantly downregulated GLB1 in the liver, showed a decreasing trend in GLB1 in the lung, heart, and spleen tissues, and significantly decreased P21 expression in the liver and lung. Fisetin treatment significantly decreased TREM2 in the lung tissues and showed a trend of downregulation in the liver, spleen, and heart. A significant decrease in NRLP3 in the liver was observed after fisetin treatment. Finally, fisetin treatment significantly downregulated SOD1 in the liver and spleen while upregulating CAT in the spleen. In conclusion, we found that senescent cells were widely present in the cerebral brain cortex and cerebellum and non-CA area of the hippocampus of old sheep. Fisetin treatment significantly decreased senescent neurons, astrocytes, and microglia in both gray and white matter of the cerebral brain cortex and non-CA area of the hippocampus. In addition, fisetin treatment decreased senescent gene expressions and inflammasomes in other organs, such as the lung and the liver. Fisetin treatment represents a promising therapeutic strategy for age-related diseases.

## 1. Introduction

Fisetin (3,7,3′,4′ tetrahydroxyflavone) is a natural flavonoid that is found in strawberries, cucumbers, apples, onions, etc. [1]. Early studies by Maher et al. showed that fisetin activated ERK and induced cAMP response element-binding protein (CREB) phosphorylation in rat hippocampus, facilitated long-term potentiation, and enhanced object recognition in mice [2]. Fisetin was found to exert neurotrophic effects by promoting rat cortical neuron survival and generating long neurites via promoting proteasome activity, not through activation of pERK or increase of glutathione [3,4]. Cumulative evidence from this research team indicates that fisetin is a novel neuroprotective and cognition-enhancing natural supplement. Fisetin not only has direct antioxidant activity, it increases the intracellular levels of glutathione (GSH), a major intracellular antioxidant, and maintains mitochondrial function in the presence of oxidative stress. In addition, fisetin has anti-inflammatory activity against microglial cells and inhibits the activity of 5-lipoxygenase, consequently reducing the production of lipid peroxides and their pro-inflammatory byproducts. This wide range of actions suggests that fisetin has the potential to reduce age-related decline in brain function [5].

Fisetin increases GSH levels mainly by activating transcription factor 4 (ATF4) under basal conditions while activating both ATF4 and NF-E2-related factor 2 (NRF2) under oxidative stress [6]. In an animal model, oral treatment with fisetin for APPswe/PS1dE9 double transgenic Alzheimer’s disease (AD) mice from 3 to 12 months of age prevented the development of learning and memory deficits [7]. This effect correlated with an increase in ERK phosphorylation and a decrease in protein carbonylation, a marker of oxidative stress [7]. In addition, fisetin decreased the levels of p25, the cyclin-dependent kinase 5 (Cdk5) activator p35 cleavage product, in both control and AD brains [7]. It has been shown that elevated levels of p25 relative to p35 cause dysregulation of Cdk5 activity, leading to neuroinflammation and neurodegeneration [7]. These effects were mediated by its anti-inflammatory effects, including changes in eicosanoid synthesis, and the maintenance of markers of synaptic function in the AD mice [7]. Fisetin was found to reduce cognitive deficits in old senescence-accelerated prone 8 (SAMP8) mice, a model for sporadic AD and dementia, while restoring multiple markers associated with impaired synaptic function, stress, and inflammation [8]. Fisetin and other flavonoids function as mitochondrial uncouplers to mitigate neurodegeneration in aged *C. elegans*, possibly via a PINK1/Parkin mitophagy process [9]. Further, fisetin enhanced mental health in the aged animals [10]. Supplementing fisetin (oral 20 mg/kg/BW/day) for four weeks improved relative electroencephalograph α-power, β-power, and multi-unit activity (MUA) count in aged rats [10]. Fisetin-treated aged rats showed significantly improved cognitive and behavioral performances compared to non-treated aged rats [10]. Further, fisetin can inhibit aggregation of the tau fragment, K18, and can disaggregate tau K18 filaments in vitro and prevent the formation of tau aggregates in cells [11].

Fisetin serves as a caloric restriction mimetic. When administrated (15 mg/kg b.w., orally) to young D-gal induced aged (D-gal 500 mg/kg b.w. subcutaneously) or naturally aged rats for 6 weeks, fisetin significantly decreased the level of pro-oxidants and increased the level of antioxidants [12]. Furthermore, fisetin ameliorated mitochondrial membrane depolarization, apoptotic cell death and impairments in the activities of synaptosomal membrane-bound ion transporters in aged rat brains. Fisetin upregulated the expression of autophagy genes (Atg-3 and Beclin-1), sirtuin-1 (Sirt-1), and neuronal markers (neuron-specific enolase (NSE) and neuroglobin (Ngb)), and downregulated the inflammatory genes (interleukin 1β (IL-1β) and tumor necrosis factor α (TNF-α)) and Sirt-2 genes in aged rat brains [12]. The same treatment regimen suppressed aging-induced increases in the levels of reactive oxygen species (ROS), eryptosis, lipid peroxidation, and protein oxidation in rat erythrocytes [13]. Fisetin prevented D-gal-mediated ROS accumulation by regulating endogenous antioxidant mechanisms, such as Sirt1/Nrf2 signaling, and suppressed the activated *p*-JNK/NF-kB pathway and its downstream targets, such as inflammatory cytokines [14]. Fisetin enhanced phosphorylation and nuclear translocation of Nrf2, which subsequently activated antioxidant enzyme heme oxygenase-1 (HO-1) in retinal pigment epithelium (RPE) cells, contributing to the amelioration of oxidative stress-induced ocular disorders [15]. A similar effect was observed in C2C12 myoblasts cells [16].

Acute or intermittent treatment of progeroid and old mice with fisetin reduced senescence markers in several tissues [17]. Fisetin reduced senescence in a subset of cells in murine and human adipose tissue [17]. Administration of fisetin to wild-type aged mice restored tissue homeostasis, reduced age-related pathology, and extended median and maximum lifespan [18]. Senescent cells accumulate with ageing; they are resistant to apoptosis and possess upregulated anti-apoptotic pathways which defend them against their own inflammatory senescence-associated secretory phenotype (SASP), allowing them to survive while killing neighboring cells [17]. Because senescent cells take weeks to reaccumulate, senolytics can be administered intermittently in a “hit-and-run” approach [17]. There may be crosstalk between fisetin’s anti-senescence role and neuroprotection [19]. Fisetin treatment has been shown to restore muscle stem cell function in muscular dystrophy mice and progeria mice by targeting senescent cells in the diseased or aged muscle tissues [20,21]. Our recent work showed that fisetin selectively attenuated markers of senescence in a dose-dependent manner while maintaining the differentiation potential of expanded human adipose stem cells in vitro [22].

Fisetin suppressed renal tubular senescence, attenuated renal fibrosis, and improved tubular repair, as indicated by restoration of tubular regeneration and renal function [23]. Fisetin treatment reduced renal fibrosis by inhibiting the phosphorylation of SMAD3, oxidative damage, inflammation, apoptotic cell death, and accumulation of profibrotic M2 macrophages in the obstructed kidneys [24]. In cultured human proximal tubular cells, fisetin treatment inhibited TGF-β1-induced phosphorylation of SMAD3 and SMAD2 [24].

Fisetin demonstrated a potent cardioprotective efficacy against Ang-II induced apoptosis in H9c2 cells and in spontaneous hypertension rat model [25]. Fisetin treatment significantly reduced the apoptotic nuclei number and apoptotic proteins such as TNF-α, Fas L, Fas associated via death domain (FADD), Cleaved caspase-3, and Cleaved poly (ADP-ribose) polymerase (Cleaved PARP) [25]. Fisetin treatment increased cell survival and anti-apoptotic proteins, including B-cell lymphoma 2 (Bcl-2), Bcl-x_L,_ phosphorylated insulin-like growth factor 1 receptor (p-IGF1R), phosphorylated phosphoinositide 3-kinases (p-PI3K), and phosphorylated protein kinase B (p-AKT), in both in vitro and in vivo models [25]. Further, fisetin treatment reduced both atherosclerosis plaques and lipid accumulation in the aortic sinus in apoE^−/−^ mice fed with a high-fat diet [26]. Fisetin has been shown to reduce the expression of proprotein convertase subtilisin/kexin type (PCSK9), lectin-like oxidized low-density lipoprotein receptor-1 (LOX-1), and other senescent markers, including p53, p21 ^CIP1/WAF1^ (p21), and P16^Ink4A^ (p16) [26]. In vascular smooth muscle cell (VSMC) cells, fisetin inhibited cellular senescence induced by the phosphatase and tensin homolog (PTEN)-PKCδ-(NADPH oxidases) NOX1-ROS signaling pathway after hydrogen peroxide (H_2_O_2_) treatment; this anti-aging effect was attributed to reduced ROS production caused by suppression of NOX1 activation [27].

Taken together, these studies demonstrate that fisetin has broad beneficial effects on age-related diseases. However, the majority of the previous studies have used mice or rats, making it difficult to extrapolate results to humans. The goal of the present study was to investigate whether systemic fisetin treatment of old sheep would reduce senescent cells in the brain and other organs.

## 2. Materials and Methods

### 2.1. Animal Use Ethics

This study was approved by the Institutional Animal Care and Use Committee (IACUC) of Colorado State University (#3159). Columbia Cross female sheep 6–7 years old were divided into two groups (N = 6/group). The fisetin group received fisetin at 100 mg/kg in DMSO and 10 mM ethanol by intravenous infusion on two consecutive days every week for 8 weeks, while the control group received vehicle intravenous infusion using the same regimen. Sheep were sacrificed at 8 weeks following treatment, and the brain and several other organs were harvested and dissected for histology or reverse transcriptive quantitative polymerase chain reaction (RT-Q-PCR) analysis.

### 2.2. Histology

Brain tissues were harvested after 8 weeks treatment, left hemispheres were dissected and fixed in neutral buffered formalin (NBF) for 9 days. The midbrain was sliced into three different levels and processed using 15% sucrose or 30% sucrose in PBS, then embedded in NEG-50 freezing medium, (Fisher Scientific, Hampton, NH, USA). The cerebellum was harvested, sliced in the middle, and embedded in NEG-50 freezing medium after 9 days of fixation. Furthermore, the hippocampus was dissected (after 6 months fixation) and processed using 15% sucrose and 30% sucrose and embedded in freezing medium and snap frozen in liquid nitrogen. The cerebral brain cortex (mid-brain level), cerebellum, and hippocampus sections were cut into slices of 8 µm thickness using Leica Cryostat for histology and immunofluorescent staining. The right hemispheres were used to collect fresh cerebral brain cortex and frozen in −80 °C for RNA isolation and RT-Q-PCR. H&E staining was performed using ANATECH extra-strength hematoxylin and eosin Y in 75% alcohol (ANATECH LTD, Battle Creek, MI, USA) following the manufacturer’s protocol.

### 2.3. Senescent-Associated β-Galactosidase (SA-β-Gal) Staining

Brain cortex and cerebellum cryosections were used to perform SA-β-Gal staining using a Senescence beta-galactosidase staining kit (#9860, Cell Signaling Technology, Danvers, MA, USA). The staining procedure followed the manufacture’s protocol. Blue staining indicated positive cells. Both intense blue staining and light blue that could be distinguished from surrounding cells were counted as positive. Positive cells were counted using Image J. Positive cell numbers in the 100× microscope field were used for comparison between groups.

### 2.4. GL13 Staining Using SenTraGor Reagent for Detection of Senescent Cells

GL13 staining for detection of lipofuscin was performed on dissected hippocampus tissue sections because SA-β-Gal staining did not work on long-term fixed tissues in NBF. GL13 staining was performed according to the protocol described by Evangelou K et al. [28] and an online protocol (https://sentragortech.com,SenTraGor_Protocol_IHC_LabSupplies.pdf, accessed on 28 July 2023). Briefly, 20 mg of SenTraGor™ Cell Senescence Reagent (Item No. 35568, Cayman Chemical, Ann Arbor, MI, USA) was dissolved in 3.75 mL absolute ethanol and incubated in 56 °C water with the lid sealed with parafilm for 120 min. The dissolved dye was kept in the original vial and sealed with parafilm to avoid evaporation of ethanol and stored at room temperature (RT) in the dark. Cryosections of dissected hippocampus were dried for 10 min and refixed with NBF for 8 min and washed twice with Tris base saline buffer (TBS) (pH = 7.4) for 5 min. Endogenous hydrogen peroxidase was inactivated in 0.5% hydrogen peroxide (H_2_O_2_) in TBS for 30 min followed by three TBS washes of 5 min for each wash. The slides were then washed in 50% ethanol and 70% ethanol for 5 min each. Two to three drops of dissolved SenTraGor dye were added to the slide using a 13 mm filter syringe, and a cover glass was immediately placed on the top of the dye to avoid evaporation. The slides were then transferred to the 50% ethanol in Coplin jar and the cover glass was gently removed. After the cover glass was removed, the washing protocol was performed four times with 50% ethanol, four times with TBS, and finally treatment with 0.5%Triton X 100 in TBS for 3 min. In this step, instead of using anti-Biotin antibody we used Vector Elite ABC kit (PK-6100, Vector Laboratories, Inc., Newark, CA, USA),) to directly detect labeled biotin on the SenTraGor reagent for 2 h at RT, then, after three rinses in TBS, DAB color reaction was performed using DAB Substrate Kit for 5 min (SK-4100, Vector Laboratories, Inc., Newark, CA, USA). The slides were finally rinsed with tap water. Hematoxylin QS Counterstain (H-3404-100, Vector Laboratories, Inc., Newark, CA, USA) was used for counterstaining nuclei for 30 s. Slides were rinsed with tap water and then running tap water for 10 min, dehydrated with gradient alcohol, cleared with two changes of xylene, and dried and mounted with Cytoseal (Fisher Scientific, Hampton, NH, USA). Images were captured at 100× using a Nikon Ti-Eclipse microscope. The number of brown stained cells in the CA and non-CA regions of the hippocampus was counted using Image J.

### 2.5. Immunofluorescence Staining

To detect which cells become senescent in the brain tissues, immunofluorescence (IF) staining for P16 and β-galactosidase (GLB1) was colocalized with the astrocyte marker glial fibrillary acidic protein (GFAP), neuronal marker NEUN, and microglial marker ionized calcium-binding adaptor molecule 1 (IBA1) in the brain cortex and dissected hippocampus. The IF procedure was performed using the same principle and procedure previously described [29]. Briefly, section slides were dried at RT and refixed in neutral buffered formalin for 8 min. Heat retrieval was performed in a pH = 6.0 citrate buffered 92 °C water bath for 20 min. For the GLB1/NEUN, GLB1/GFAP, and GLB1/IBA1 double IF, we used pH = 9 Tris-EDTA buffer for heat-mediated antigen retrieval. After antigen retrieval, slides were washed with phosphate buffered saline (PBS) three times and blocked with 5% donkey serum (Jackson ImmunoResearch Labroratory Inc., West Grove, PA, USA) at ambient temperature for 1 h. Then, primary antibodies diluted in 5% donkey serum were added to the sections and incubated at 4 °C overnight. The primary antibody dilutions were as follows: rabbit anti-GFAP (ab68428, 1:100, Abcam, Cambridge, United Kingdom)), rabbit anti-IBA1(10904-1-AP, 1:200 dilution, Proteintech, Rosemont, IL, USA) ), rabbit anti-NEUN (ab177487, 1:2000, Abcam, Cambridge, United Kingdom), mouse anti-P16 (SC1661, 1:50, Santa Cruz Biotechnology, Dallas, TX, USA)), and mouse anti-GLB1 (66586-1-Ig, 1:200,Proteintech, Rosemont, IL, USA). For dissected hippocampus staining, we used mouse anti-P16 from ThermoFisher Scientific (MA5-17142, 1:400, Waltham, MA, USA) because the anti-P16 antibody from Santa-Cruz Biotechnology did not work for hippocampus sections. After incubation with the primary antibodies, slides were washed three times with PBS. The sections were then incubated with donkey anti-rabbit-594 (711-585-152,Jackson ImmunoResearch Laboratory Inc., West Grove, PA, USA) and donkey anti-mouse 488 (715-545-150, Jackson ImmunoResearch Laboratory Inc., West Grove, PA, USA) at 1:200 dilution and incubated at ambient temperature for 2 h. After incubation with the secondary antibodies, the slides were washed three times and nuclei were stained with 4′,6-diamidino-2-phenylindole (DAPI, ThermoFisher Scientific, Waltham, MA, USA) at 1 mg/mL in PBS for 10 min. Slides were finally washed with PBS and deionized water before being mounted with VectaMount^®^ AQ Aqueous Mounting Medium (H-5501-60, Vector Laboratories, Inc., Newark, CA, USA). Immunofluorescence images were captured using a Nikon Ti microscope at 200X magnification and cell numbers were quantified with Image J software (Ij153.Java 8, https://imagej.nih.gov/ij/download.html (accessed on 28 July 2023)).

### 2.6. Brain Cortex, Heart, Spleen, Lung, Liver Tissue, and Bone Marrow Harvesting and Homogenization

Brain cortex tissues from the same location were cut, snap-frozen in liquid nitrogen, and subsequently stored at −80 °C until RNA isolation. The left ventricle of the heart was cut at the same location, snap-frozen in liquid nitrogen and stored at −80 °C until RNA-isolation and RT-Q-PCR analysis. Spleen, liver, and lung tissues from each sheep were cut and frozen at −80 °C until RNA extraction. Briefly, tissues were rinsed with PBS and around 0.5 g of tissues were transferred to 2 mL tubes, cut with scissors to approximately 1 mm size, and then homogenized with an IKA-T10 tissue dispenser in 2 mL TRIzol™ Reagent (Cat# 15596026, ThermoFisher Scientific, Waltham, MA, USA). Tissues were homogenized using 30 s of homogenization and then placed on ice for 30 s. After homogenization, tissue lysates were centrifuged at 12,000× *g* for 5 min to remove fat or tissue debris. The supernatants were transferred to a new tube for RNA isolation following the manufacturer’s protocol. Furthermore, bone marrow was aspirated from the sheep iliac crest and nucleated cells were isolated using Percoll gradient reagent for RNA extraction using TRIzol reagent as indicated below.

### 2.7. RNA Isolation, cDNA Synthesis, and Semi-Quantitative and Quantitative-PCR (Q-PCR)

All RNAs were isolated using the TRIzol reagent protocol provided by the manufacturer (#15596026, ThermoFisher Scientific, Waltham, MA, USA). Total RNA was air dried for 7 min, resuspended in DNase/RNase free water using appropriate volume based on the RNA pellet size, and dissolved for 10 min at RT. RNA concentration was measured with a Nanodrop plate equipped in Tecan Plate Reader (Tecan Trading AG, Seestrasse 103, 8708 Männedorf, Switzerland). First-strand cDNA synthesis was then conducted using iScript™ Reverse Transcription Supermix, 100 × 20 µL rxns, 400 µL (1708841, Bio-Rad Laboratories, Hercules, CA, USA ) in 20 µL reaction using 25 °C for 5 min, 46 °C for 30 min and 95 °C for 1 min. After cDNA synthesis, cDNA was diluted using DNase/RNase free H_2_O to 10 ng/ µL. Subsequently, semi-quantitative or Q-PCR was performed. For the brain tissues, we performed both semi-quantitative-PCR and Q-PCR. Semi-quantitative PCR was performed using GoTaq^®^ G2 Master Mixes (M7823, Promega Corporation, Madison, WI, USA). PCR products were electrophoresed using 1.5% agarose gel (Bio-Rad Laboratories, Hercules, CA, USA) and gel images were captured using the ChemiDoc MP System (Bio-Rad Laboratories, Hercules, CA, USA). Housekeeping gene and target band density were quantified with Image Lab software (Bio-Rad Laboratories, Hercules, CA, USA). Q-PCR was performed using SsoAdvanced Universal SYBR^®^ Green Supermix, 1000 × 20 µL rxns, 10 mL (10 × 1 mL) (Cat# 1725272, Bio-Rad Laboratories, Hercules, CA, USA) using 10 µL reaction with 1 µL diluted cDNA. Q-PCR was conducted on an ABI system. The analyzed genes included Glyceraldehyde 3-phosphate dehydrogenase (GAPDH), GFAP, lysosomal β-galactosidase (GLB1), super oxide dismutase 1 (SOD1), catalase (CAT), neurofilament heavy chain (NEFH), neurofilament light chain (NEFL), interleukin 10 (IL10), interleukin 8 (IL8), P21, P53, NOD-, LRR-, and pyrin domain-containing protein 3 (NLRP3), and Triggering Receptor Expressed on Myeloid Cells 2 (TREM2). Gene expression levels were expressed using fold change of control (2^−∆∆CT^) methods. All primers were designed using primer 3 input free online software [30,31]. Primer information is shown in Table 1.

### 2.8. Plasma Malondialdehyde (MDA) and S100 Calcium-Binding Protein B (S100B) Measurement

Prior to sacrifice (8 weeks post-treatment), peripheral blood was collected from the sheep. Plasma was isolated by centrifugation. MDA was measured using a Lipid Peroxidation Assay (MDA) (Colorimetric/Fluorometric) color reaction kit (ab118970, Abcam, Cambridge, United Kingdom) following the manufacturer’s protocol. S100B was measured using a Sheep S100B (S100 Calcium Binding Protein B) ELISA Kit (Cat#: orb1100745, Biorbyt, Cambridge, United Kingdom) using the manufacturer’s manual.

### 2.9. Statistical Analysis

All data were analyzed with two-group *t*-tests using GraphPad Prism 9.5.0 (*p* ≤ 0.05 was considered statistically significant.

## 3. Results

### 3.1. Effects of Fisetin Treatment on the General Morphology of Old Sheep Brains

**Figure 1:**
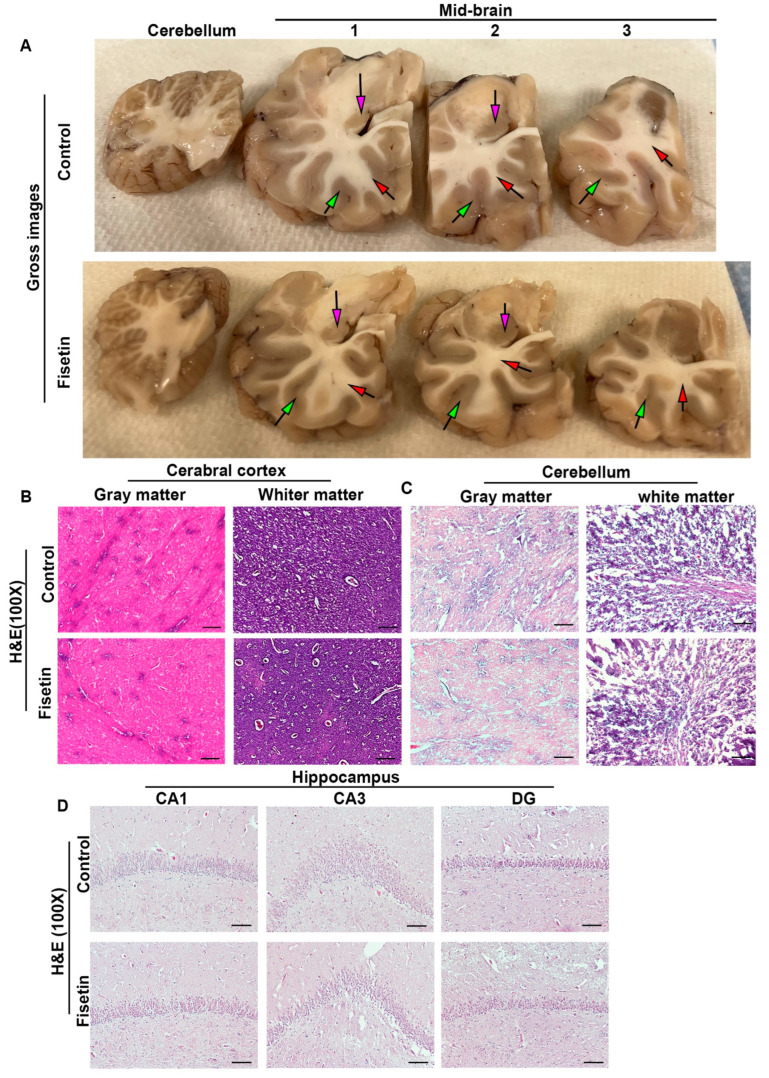
Gross images and H&E staining of brain tissues after fisetin treatment. (**A**) Cross-sectional view of the cerebellum and left hemisphere of cerebral brain cortex at different axial level. Red arrows indicate white matter, green arrows indicate gray matter, and pink arrows point to hippocampus. (**B**) H&E staining of cerebral cortex. (**C**) H&E staining of cerebellum for gray matter and white matter, showing different cellularity. (**D**) H&E staining of hippocampus at CA1, CA3, and DG areas. Fisetin treatment did not significantly affect the general morphology of different regions. Scale bars = 100 µm.

Gross images showed no obvious differences on the cerebral brain cortex (midbrain) between fisetin-treated sheep and vehicle-treated sheep (Figure 1A). H&E staining did not reveal any significant differences between fisetin-treated and vehicle-treated sheep brain for the cerebral cortex (Figure 1B), cerebellum (Figure 1C), or different regions of the hippocampus (Figure 1D). These results indicate that fisetin treatment did not significantly affect the general morphology of different brain regions.

### 3.2. Effecta of Fisetin Treatment on the Cellular Senescence of Different Brain Regions of Old Sheep

**Figure 2:**
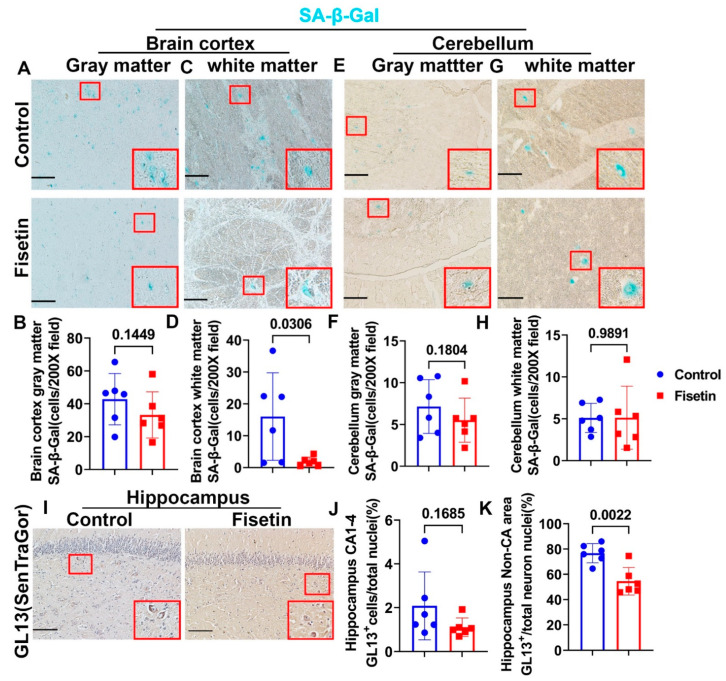
Effects of fisetin treatment on senescent cells in different regions of the brain. (**A**) Brain cortex SA-β-Gal staining of gray matter region showing many blue stained cells that are mainly large neurons. Nuclei were not stained. (**B**) Quantification of SA-β-Gal-positive cells. (**C**) Brain cortex SA-β-Gal staining in the white matter region. (**D**) Quantification of SA-β-Gal-positive cells in the brain cortex white matter. (**E**) Cerebellum gray matter SA-β-Gal staining. (**F**) Quantification of SA-β-Gal-positive cells in the cerebellum gray matter. (**G**) Cerebellum white matter SA-β-Gal staining. (**H**) Quantification of SA-β-Gal-positive cells in the white matter of the cerebellum. (**I**) GL13 (SenTraGor) staining for lipofuscin. GL13^+^ cells were stained brown in the cytoplasm. (**J**) Quantification of GL13^+^ cells in the CA area neurons. (**K**) Quantification of GL13^+^ cells of large neurons in non-CA area of hippocampus. Red boxes in each image highlighted SA-β-Gal-positive cells or GL13^+^ cells. Scale bars = 100 µm. Exact *p* values are indicated between group bars.

We found many SA-β-Gal positive cells in the gray matter of the control sheep’s cerebral brain cortex tissues, which indicated the presence of senescent cells in 6–7 years old sheep (blue staining; the insets highlight positive cells). These positive cells were mainly large neurons with nuclei that were not stained because SA-β-Gal only located in lysosomes (Figure 2A). Fisetin treatment showed a decreasing trend of SA-β-Gal-positive cells compared to control group (*p* = 0.1449), but many SA-β-Gal positive cells were still present after fisetin treatment (Figure 2B). We found many SA-β-Gal-positive cells in the white matter of the cerebral brain cortex, but relative fewer compared to the gray matter. These SA-β-Gal-positive cells were also mostly neurons. Fisetin treatment significantly decreased the number of SA-β-Gal-positive cells in white matter compared to control (*p* = 0.0306, Figure 2C,D). Furthermore, we found SA-β-Gal-positive cells in the gray matter of the cerebellum, but not as many as in the brain cortex region. These positive cells were also mainly large neurons (Figure 2E). Fisetin treatment showed a decreasing trend of SA-β-Gal-positive cells compared to control group (*p* = 0.1804, Figure 2F). Further, SA-β-Gal-positive cells were found in the white matter of the cerebellum, and these were also mainly large neurons (Figure 2G). Fisetin treatment did not significantly decrease the number of SA-β-Gal-positive cells in the white matter of the cerebellum (Figure 2H). In addition, GL13 staining of the hippocampus using SenTraGor reagent demonstrated that GL13^+^ senescent cells were mainly large neurons in the non-CA region of hippocampus. Few GL13^+^ senescent cells were found in the neurons of CA1-4 area (Figure 2I, red boxes highlighted brown positive cells). Only 1–2% of CA1-4 area neurons were positive for GL13 staining. Fisetin treatment showed a decreasing trend of GL13^+^ cells in the CA1-4 area (Figure 2J) (*p* = 0.1685). However, fisetin treatment significantly reduced the number of GL13^+^ large neurons in the non-CA area of hippocampus (Figure 2K) (*p* = 0.0022). Taken together, these results demonstrate the presence of senescent cells in different regions of the brain and the ability of fisetin to decrease the number of senescent cells in the brain.

### 3.3. Effects of Fisetin Treatment on Cellular Senescence in Neurons, Astrocytes, and Microglial Cells of the Cerebral Cortex

**Figure 3:**
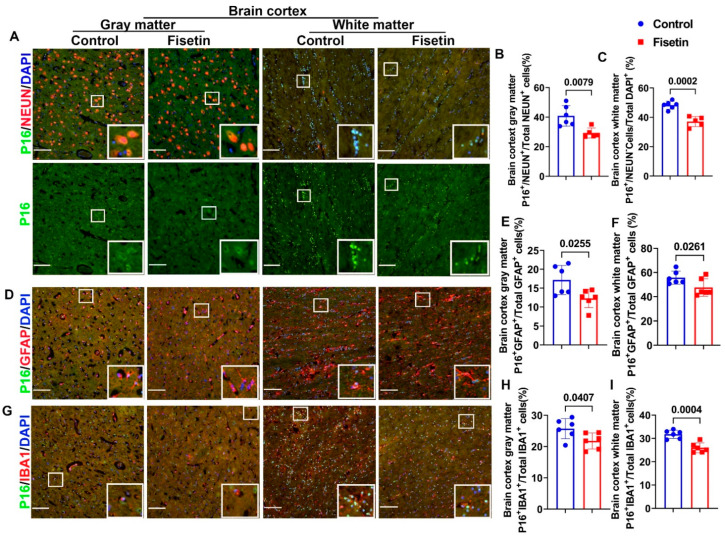
Identification of senescent cells of brain cortex and effects of fisetin treatment. (**A**) P16 and NEUN double immunofluorescent staining for neurons in brain cortex gray and white matter. P16 is stained green in the nuclei. NEUN-labeled neurons are stained in red in the nuclei and perinuclear cytoplasm. Nuclei are stained in blue with DAPI. There are many large red-stained neurons in the gray matter and very few in the white matter. The green channel reveals P16^+^ cells in the gray matter that are bigger with diffused staining, while there are small cells with strong signals in the white matter. (**B**) Quantification of P16^+^/NEUN^+^/Total NEUN^+^ cells percentage in the gray matter. (**C**) Quantification of P16^+^/NEUN^−^/Total DAPI^+^ cells in the white matter. (**D**) P16 and GFAP double immunofluorescence staining of astrocytes in the brain cortex gray and white matter. P16^+^ cells are stained green in the nuclei, while GFAP^+^ cells are stained red for astrocytes with many projections. (**E**) Quantification of P16^+^/GFAP^+^/Total GFAP^+^ cells percentage in the gray matter. (**F**) Quantification of P16^+^/GFAP^+^/Total GFAP^+^ cells percentage in the white matter. (**G**) P16 and IBA1 double immunofluorescence staining for microglia cells. P16^+^ cells are stained green in the nuclei, while IBA1^+^microglia cells are stained in red. (**H**) Quantification of P16^+^/IBA1^+^/Total IBA1^+^cells percentage in the gray matter. (**I**) Quantification of P16^+^/IBA1^+^/Total IBA1^+^ cells percentage in the white matter. s White boxes highlight positive cells. Scale bars = 100 µm. Exact *p* values are shown between the group bars.

Because we found many senescent cells in the old sheep, both in the cerebral brain cortex and cerebellum, we wanted to identify which brain cells became senescent and whether fisetin treatment decreased the senescent cell burden. We first performed P16 staining, an established marker of senescent cells, colocalized with different neuronal cell markers to identify which cells became senescent. By colocalization of P16 with NEUN, a neuronal marker, we found many NEUN-positive red stained cells in the gray matter, while P16 staining was localized in the nuclei of both neurons and non-neurons. P16 staining in the neurons was relatively diffuse due to the loose nuclear structure of neurons (green channel). Colocalized cells appeared orange or yellow in color (Figure 3A, gray matter). Quantification indicated that fisetin treatment significantly decreased P16^+^/NEUN^+^ cells/Total NEUN^+^ cells percentage in NEUN^+^ neurons in the brain cortex gray matter (*p* = 0.0079) (Figure 3B). Furthermore, very few NEUN^+^ cells were detected in the white matter, while many small P16^+^ green cells that were not colocalized with NEUN were detected (Figure 3A, white matter). Therefore, we counted the P16^+^ NEUN^−^cells/Total DAPI cells percentage. Fisetin treatment significantly decreased the P16^+^ NEUN^−^cells/Total DAPI cells percentage in the white matter (*p* = 0.0002) (Figure 3C). We further performed P16 staining colocalized with astrocyte marker GFAP. GFAP^+^ astrocytes were stained red, showing many projections with small cytoplasm in the gray matter (Figure 3D, gray matter). Fisetin treatment significantly reduced the P16^+^/GFAP^+^/Total GFAP^+^ cells percentage of astrocytes in the gray matter (*p* = 0.0255) (Figure 3E). In the white matter, many more GFAP^+^ cells were detected (Figure 3D white matter). Fisetin treatment significantly decreased P16^+^/GFAP^+^/Total GFAP^+^ cells percentage of astrocytes in the white matter (*p* = 0.0261) (Figure 3F). Additionally, we colocalized P16 staining with the microglial marker IBA1. IBA1^+^ cells were stained in red, showing small cells with few projections; a few IBA1^+^ cells were colocalized with P16 (Figure 3G gray matter). Fisetin treatment significantly decreased P16^+^/IBA1^+^/Total IBA1^+^ cells percentage in the microglia cells (*p* = 0.0407) (Figure 3H). Furthermore, in the white matter, many more IBA1^+^ (red) and P16^+^ (green) small cells with some colocalization were found (Figure 3G white matter). Fisetin treatment significantly reduced the P16^+^/IBA1^+^/Total IBA1^+^ percentage in microglia cells in the white matter (*p* = 0.0004) (Figure 3I).

**Figure 4:**
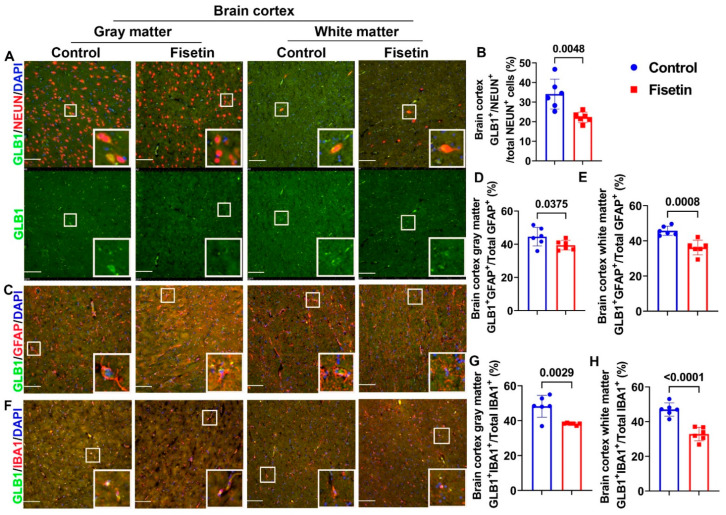
Identification of senescent cells in the brain cortex using double IF staining of GLB1 with different neuronal markers. (**A**) Double IF of GLB1 with NEUN. NEUN^+^ neurons are stained in red in the nuclei and perinuclear cytoplasm, while GLB1 is stained in green in the cytoplasm. Nuclei are stained in blue with DAPI. Colocalized cells are stained in orange/yellow. The green channel shows GLB1 staining. (**B**) Quantification of GLB1^+^NEUN^+^/Total NEUN^+^ cells percentage in the gray matter of the brain cortex. (**C**) Double IF of GLB1 with GFAP. GFAP^+^ cells are stained in red, showing many projections. GLB1^+^GFAP^+^cells are shown in orange, highlighted in the insets. (**D**) Quantification of GLB1^+^GFAP^+^/Total GFAP^+^ cells percentage in the gray matter of the brain cortex. (**E**) Quantification of GLB1^+^GFAP^+^/Total GFAP^+^ cells percentage in the white matter of the brain cortex. (**F**) Double IF for GLB1 and IBA1. IBA1^+^ cells are stained in red color, and are small with few projections. (**G**) Quantification of the GLB1^+^IBA1^+^/total IBA1^+^ cells percentage in the gray matter of the brain cortex. (**H**) Quantification of the GLB1^+^IBA1^+^/total IBA1^+^ cells percentage in the white matter of the brain cortex. Scale bars = 100 µm. White boxes in each image highlight positive cells. Exact *p* values are shown between the group bars.

Furthermore, although P16 is an established marker for cellular senescence, not all P16^+^ cells are senescent cells [32]; thus, we subsequently performed IF staining for an additional senescent cell marker, GLB1, and assessed colocalization with different neuronal markers. We found many NEUN^+^ neurons in the gray matter, where some neurons were GLB1^+^NEUN^+^ double positive cells (Figure 4A gray matter, highlighted in white boxes). We found very few NEUN^+^ cells in the white matter, and some were colocalized with GLB1. Fisetin treatment significantly decreased GLB1^+^/NEUN^+^ /Total NEUN^+^ cells percentage in the gray matter of the brain cortex (Figure 4B) (*p* = 0.0048). We also performed GLB1 and GFAP double IF to detect senescent astrocytes. We found GLB1^+^GFAP^+^ cells in the gray and white matter of both the control and fisetin groups (Figure 4C and insets). Fisetin treatment significantly reduced the GLB1^+^GFAP^+^/total GFAP^+^ cell percentage in both the gray (*p* = 0.0375) and white matter (*p* = 0.0008) of the brain cortex (Figure 4D,E). Furthermore, we performed double IF for GLB1 and IBA1 to detect senescent microglia, revealing GLB1^+^IBA1^+^ cells in both the gray and white matter of the brain cortex (Figure 4F, highlighted in white boxes). Fisetin treatment significantly decreased the GLB1^+^IBA1^+^/total IBA1^+^ cells percentage in both the gray matter (*p* = 0.0029) and white matter (*p* < 0.0001) of the brain cortex (Figure 4G,H). These data, taken together, indicate that fisetin treatment significantly decreases senescent neurons, astrocytes, and microglia.

### 3.4. Effects of Fisetin Treatments on Cellular Senescence of Neurons, Astrocytes, and Microglial Cells of the Hippocampus

**Figure 5:**
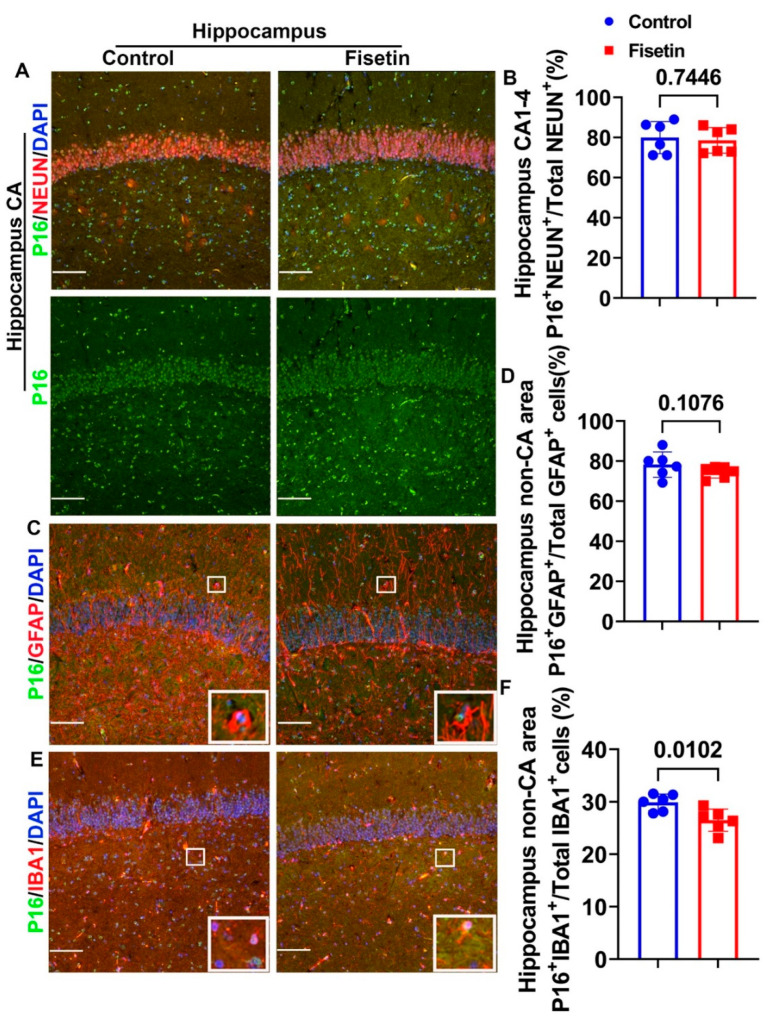
Effects of fisetin treatment on the cellular senescence of different cells in the hippocampus. (**A**) NEUN and P16 double immunofluorescence staining for the hippocampus, focusing on the CA area. NEUN^+^ cells are stained in red and many are colocalized with P16 (merged channel). P16^+^ cells are stained in green in the nuclei (green channel). (**B**) Quantification of P16^+^/NEUN^+^/Total NEUN^+^ cells in the CA1-4 area. (**C**) GFAP and P16 double immunofluorescence staining in the hippocampus. GFAP^+^ cells are stained in red, showing many projections, and are mainly located in the non-CA area. P16^+^ cells are stained in green in the nuclei. (**D**) Quantification of P16^+^GFAP^+^/Total GFAP^+^ cells in the non-CA area of the hippocampus. (**E**) IBA1 and P16 double immunofluorescence staining in the hippocampus. IBA1^+^ cells are stained in red and mainly located in the non-CA area. P16^+^ cells are stained in green in the nuclei. (**F**) Quantification of P16^+^/IBA1^+^/Total IBA1^+^ cells in the non-CA area of the hippocampus. White boxes in each image highlight positive cells of each staining. Scale bars = 100 µm. Exact *p* values are indicated between the group bars.

To determine whether fisetin treatment affects cellular senescence in the hippocampus, we dissected hippocampus from the cerebral brain cortex after fixation, processed it with PBS containing 15–30% sucrose in PBS, and embedded it in NEG-50 freezing medium. Then, 8 µm cryosections were cut for double immunofluorescence staining. First, we performed NEUN and P16 (MA5 17142, ThermoFisher Scientific) double immunofluorescence staining. NEUN/P16 double staining showed strong NEUN^+^ staining in a very organized manner in the Cornu Ammonis (CA), while large NEUN^+^ cells were scattered in the non-CA area of the hippocampus (Figure 5A, merged images). P16 was stained in green in the nuclei (Figure 5A green channel). A large number of NEUN^+^/P16^+^cells (orange or yellow colocalized cells) were detected in the CA area in both the control and fisetin groups (Figure 5A merged channel). Fisetin treatment did not significantly change P16^+^NEUN^+^/Total NEUN^+^ cells percentage in the CA area (Figure 5B). We performed double staining of P16 and GFAP to detect senescent astrocytes in the hippocampus. GFAP^+^ cells were stained in red color, showing many red projections, and were mostly located in the non-CA area, with some processes penetrating the CA area (Figure 5C, white boxes highlighted double positive cells). Therefore, we counted P16^+^/GFAP^+^ cells in the non-CA area. Fisetin treatment showed a decreasing trend of P16^+^/GFAP^+^/Total GFAP^+^ cells percentage in the non-CA area of the hippocampus (*p* = 0.1076) (Figure 5D). Furthermore, we performed double immunofluorescent staining of IBA1 and P16 to detect senescent microglia cells in the hippocampus. IBA1^+^ cells were stained in red in the small cells, showing few projections. P16^+^ cells were stained in green in the nuclei. We counted P16^+^/IBA1^+^ cells in the non-CA area because IBA1^+^ cells are not expressed by neurons in the CA area (Figure 5E, white boxes highlighted positive cells). Fisetin treatment significantly decreased P16^+^IBA1^+^/Total IBA1^+^ cells percentage in the microglia cells in the non-CA area of the hippocampus (*p* = 0.0102) (Figure 5F).

**Figure 6:**
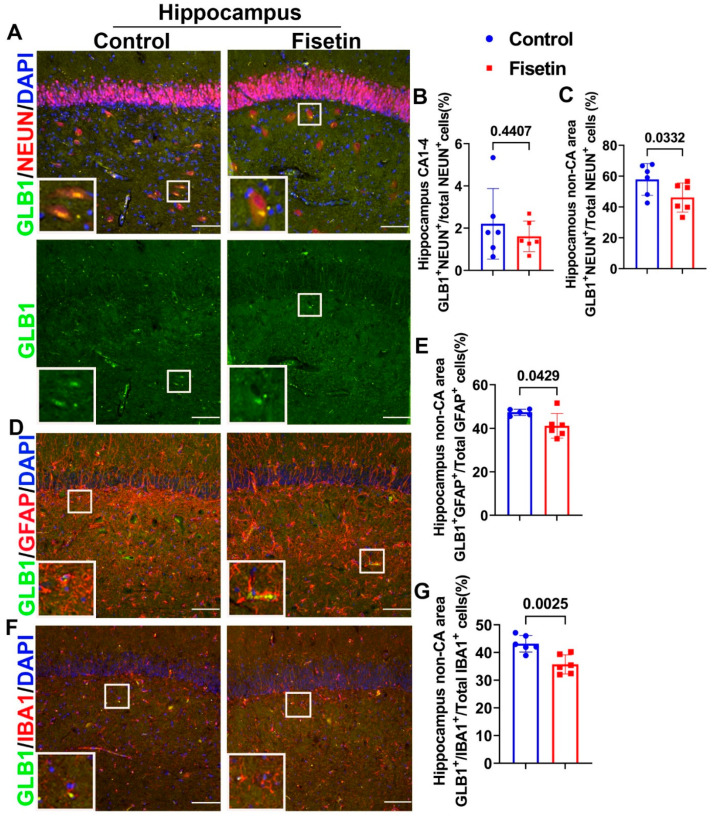
Double IF of GLB1 and neuronal marker for the hippocampus and the effects of fisetin. (**A**) Double IF for GLB1 and NEUN to detect senescent neurons. GLB1^+^ cells are stained in green in the cytoplasm (green channel). Very few GLB1^+^NEUN^+^ cells are found in the neurons of the CA 1-4 area. Many GLB1^+^NEUN^+^ cells can be observed in the non-CA area neurons, shown in a distinct patched cytoplasm yellow color (highlighted in the insets). (**B**) Quantification of GLB1^+^NEUN^+^ cells/total NEUN^+^ cells percentage in the CA1-4 area. (**C**) Quantification of GLB1^+^NEUN^+^/total NEUN^+^ cells in the non-CA area (large neurons). (**D**) Double IF for GLB1 and GFAP. GFAP^+^ astrocytes are stained in red, showing several projections in the non-CA area. GLB1^+^ cells are stained green in the cytoplasm. GLB1^+^GAFP^+^ cells are highlighted in the insets. (**E**) Quantification of GLB1^+^GFAP^+^/total GFAP^+^ cells percentage. (**F**) Double IF staining of GLB1 and IBA1 to detect senescent microglia. IBA1^+^ cells are stained in red, with smaller size and few projections in the non-CA area. GLB1^+^IBA1^+^ cells are highlighted in the insets. (**G**) Quantification of GLB1^+^IBA1^+^/total IBA1^+^ cells percentage in non-CA area. Scale bars = 100 µm. White boxes highlight double positive cells in each staining. Exact *p* values are indicated between the group bars.

To further validate whether fisetin treatment decreased senescent cells of different neuronal lineages in the hippocampus, we performed double IF of GLB1 and colocalized with NEUN, GFAP, and IBA1. We found very few GLB1^+^NEUN^+^ cells in the CA1-4 area of the hippocampus, but observed obvious large neurons with distinct patch-like yellow staining (Figure 6A merged and green channel, white boxes highlighted positive cells). The GLB1^+^NEUN^+^/total NEUN^+^ cells in CA1-4 area were less than 2% in both groups. Fisetin treatment did not change the GLB1^+^NEUN^+^/total NEUN^+^ cells percentage in CA1-4 area (Figure 6B). However, Fisetin treatment significantly reduced the GLB1^+^NEUN^+^/total NEUN^+^ cells percentage in the non-CA area (*p* = 0.0332) (Figure 6C). Double IF for GLB1 and GFAP indicated the presence of GLB1^+^GFAP^+^ cells in the non-CA area (Figure 6D, white boxes highlighted double positive cells). Fisetin treatment significantly decreased the GLB1^+^GFAP^+^/total GFAP^+^ cells percentage in the non-CA area of the hippocampus (Figure 6E) (*p* = 0.0429). In addition, GLB1^+^IBA1^+^ microglia were detected in the non-CA area of hippocampus with smaller size (Figure 6F, white boxes highlighted double positive cells). Fisetin treatment significantly decreased the GLB1^+^IBA1^+^/total GLB1^+^ cells percentage in the non-CA area of hippocampus (Figure 6G) (*p* = 0.0025). Taken together, these results indicate that although most neurons in the CA1-4 area expressed P16, very few expressed GLB1, which indicates that they did not become senescent at this age. However, many large neurons in the non-CA area expressed GLB1. Fisetin treatment significantly decreased the GLB1^+^ neurons, astrocytes, and microglia in the non-CA area of the hippocampus. These results indicate that fisetin treatment significantly reduced P16^+^ and GLB1^+^ astrocytes, microglia, and neurons in the non-CA area of the hippocampus.

Altogether, we found that fisetin treatment decreased the number of senescent cells in different regions of the brain, although to a different extent. The results are summarized in Table 2.

### 3.5. Effects of Fisetin Treatment on Gene Expression of Sheep Brain Cortex

**Figure 7:**
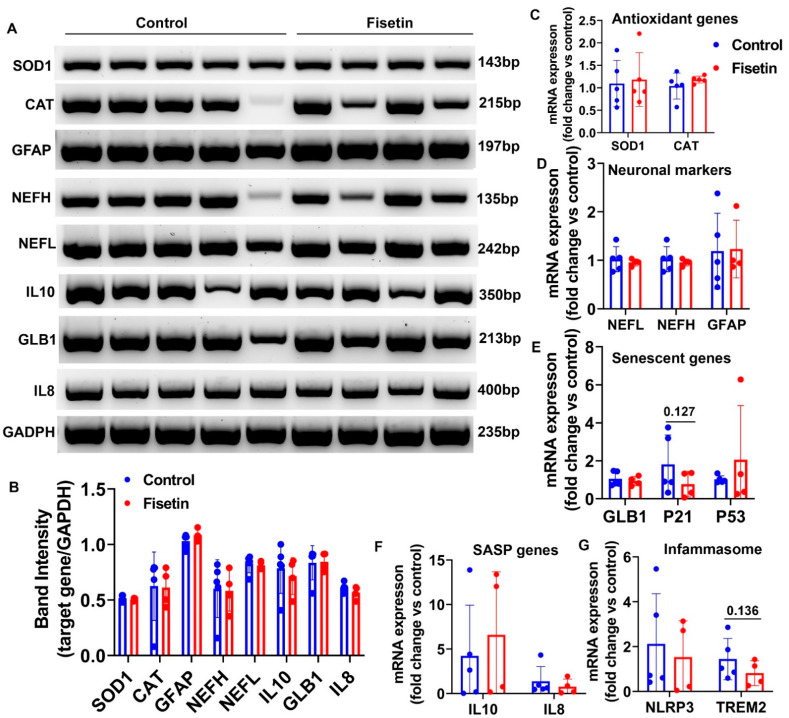
Semi-quantitative and quantitative RT- Q-PCR results in the brain cortex after fisetin treatment. (**A**) Electrophoresis images of different target genes. (**B**) Band density quantification relative to GAPDH. (**C**) RT-Q-PCR analysis of antioxidant genes. (**D**) RT-Q-PCR analysis of NEFL, NEFH, and GFAP. (**E**) RT-Q-PCR analysis of senescence related genes. (**F**) RT-Q-PCR analysis of SASP genes. (**G**) RT-Q-PCR analysis of inflammasome genes. Exact *p* values are shown between the group bars.

Semi-quantitative-PCR analyses indicated that fisetin treatment did not significantly change gene expression of antioxidant genes SOD1 and CAT, astrocyte marker (GFAP), neuronal markers (NEFH, NEFL), SASP markers interleukin 10 and 8 (IL10, IL8), or senescent marker gene GLB1 in the brain cortex (Figure 7A,B). We further performed Q-PCR analysis, finding that fisetin treatment did not significantly affect SOD1 and CAT expression in the cerebral brain cortex (Figure 7C). Fisetin treatment did not affect the neuronal markers NEFL and NEFH or the astrocyte marker GFAP (Figure 7D). Fisetin treatment did not significantly change GLB1 gene expression or P53 expression, but showed a decreasing trend of P21 expression (Figure 7E, *p* = 0.127). Fisetin treatment did not significantly affect SASP gene IL10 and IL8 expression (Figure 7F). Furthermore, fisetin treatment did not significantly affect inflammasome gene NLRP3, though it showed a decreasing trend of TREM2, another inflammasome gene (Figure 7G). In summary, fisetin treatment showed a decreasing trend of P21 and TREM2 in the brain cortex.

### 3.6. Effect of Fisetin Treatment on Gene Expression in Heart Tissues

**Figure 8:**
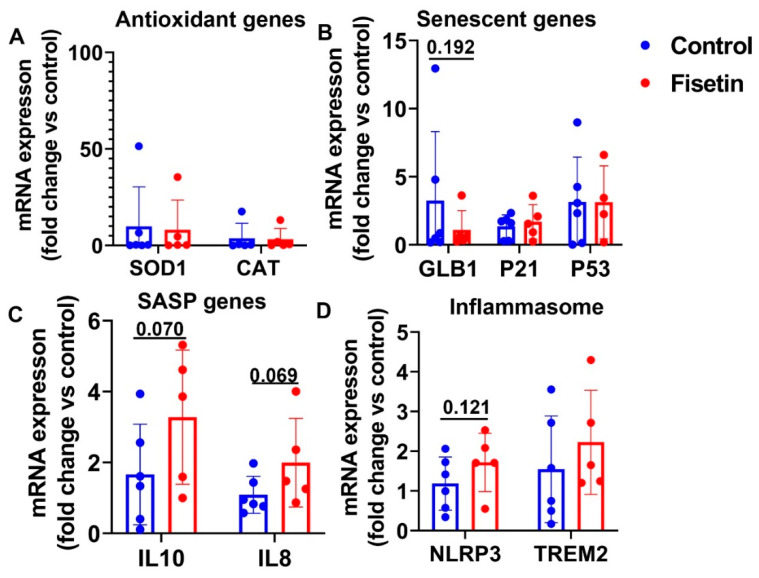
RT-Q-PCR analysis of gene expression of sheep heart tissues after fisetin treatment: (**A**) RT-Q-PCR results of antioxidant genes; (**B**) RT-Q-PCR analysis of senescence related genes; (**C**) mRNA expression of SASP genes; (**D**) mRNA expression of inflammasome genes. Exact *p* values are shown between the group bars.

To further investigate whether fisetin treatment affects gene expression of theheart, we harvested ventricle muscle of heart tissues and performed Q-PCR analysis. We found that systemic fisetin treatment did not significantly change gene expression of SOD1 or CAT in the sheep’s heart ventricle (Figure 8A). Among the senescence-related genes, we found that fisetin showed a trend of downregulating GLB1 (*p* = 0.192), without affecting P21 and P53 expression (Figure 8B). Furthermore, fisetin treatment demonstrated a trend of increasing IL10 (*p* = 0.0695) and IL8 expression (*p* = 0.0691) (Figure 8C). Finally, fisetin treatment showed a trend of upregulating the inflammasome gene NLRP3, with no effect on TREM2 expression in heart tissues (Figure 8D). Taken together, fisetin treatment showed a downregulating trend of GLB1 and upregulating trend of IL10, IL8, and NLRP3 in the heart tissues.

### 3.7. Effects of Fisetin Treatment on Sheep Spleen Tissue Gene Expression

**Figure 9:**
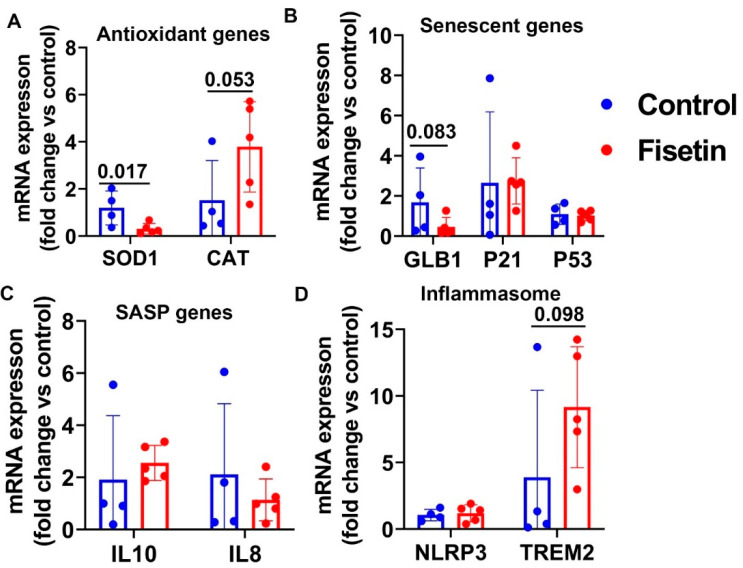
Gene expressions in the spleen tissues after fisetin treatment: (**A**) ntioxidant genes mRNA expression; (**B**) senescence-related genes mRNA expression; (**C**) SASP gene mRNA expression; (**D**) inflammasome gene expression. Exact *p* values are shown between the group bars.

We harvested spleen tissues, an important immune organ, and extracted total RNA for gene expression analysis. We found that SOD1 was significantly decreased by fisetin treatment (*p* = 0.017) and that CAT was increased (*p* = 0.053) (Figure 9A). Our results indicate that fisetin treatment showed a decreasing trend of the senescence cell marker gene GLB1 in the spleen (*p* = 0.083) (Figure 9B). No significant changes were found for P21 and P53 mRNA expression in the spleen tissues (Figure 9B). We did not find significant changes for the SASP genes IL10 and IL8 in the spleen (Figure 9C). Fisetin treatment did not significantly change the expression of NLRP3, though an increasing trend of TREM2 expression was observed (*p* = 0.098) (Figure 9D). In summary, fisetin treatment significantly decreased SOD1 expression in spleen tissues, while a decreasing trend was observed for GLB1 expression and an increasing trend for CAT expression.

### 3.8. Effects of Fisetin Treatment on Gene Expression of Bone Marrow Nucleated Cells

**Figure 10:**
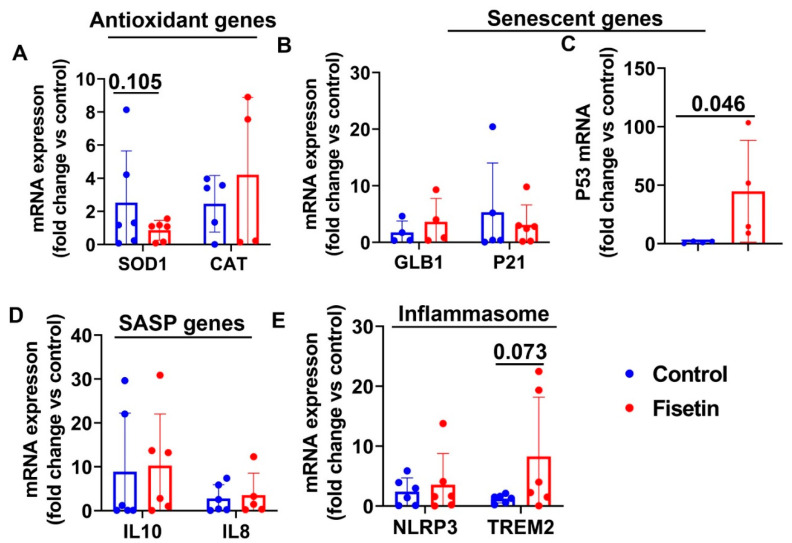
mRNA gene expression of sheep bone marrow after fisetin treatment: (**A**) antioxidant genes mRNA expression; (**B**) senescence-related genes; (**C**) mRNA expression of P53; (**D**) SASP genes; (**E**) inflammasome genes. Exact *p* values are shown between the group bars.

Furthermore, we isolated bone marrow nucleated cells from the sheep iliac crest and performed Q-PCR to investigate whether fisetin treatment affects cellular senescence in the bone marrow. Fisetin treatment showed a decreasing trend of SOD1 expression without affecting CAT expression (Figure 10A). We did not observe significant differences between fisetin treatment and control for GLB1 and P21 expression (Figure 10B). However, our results indicate that fisetin treatment significantly increased P53 expression in the bone marrow (*p* = 0.046) (Figure 10C). No statistical significance was found for IL10 and IL8 expression between fisetin treatment and control in bone marrow (Figure 10D). Additionally, fisetin treatment did not change inflammasome gene expression level of NLRP3,but showed an increasing trend of TREM2 expression (*p* = 0.073) (Figure 10E). Taken together, fisetin treatment significantly increased P53 expression, and showed a decreasing trend of SOD1 expression and an increasing trend of TREM2 expression in bone marrow.

### 3.9. Effects of Fisetin Treatment on Cellular Gene Expression in Lung Tissues

**Figure 11:**
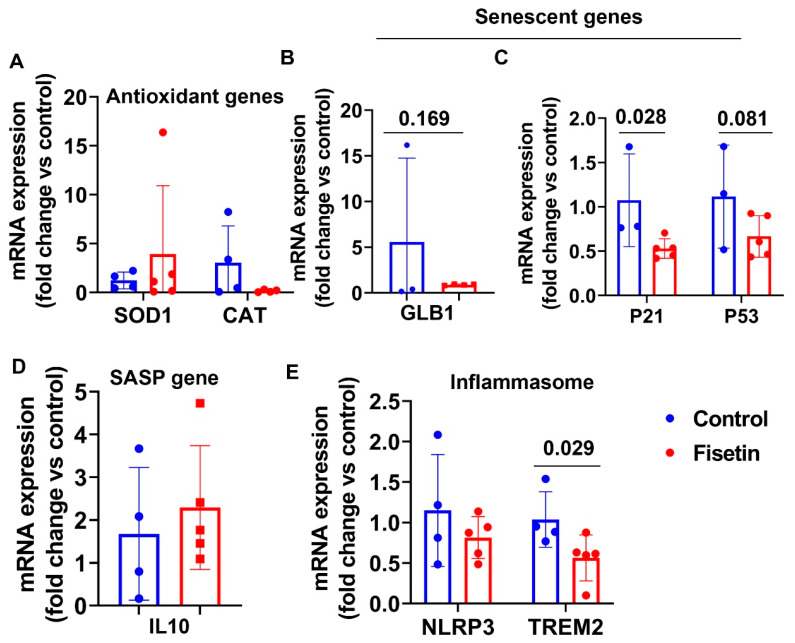
Effects of fisetin treatment on gene expression in the lung tissue: (**A**) mRNA expression of antioxidant genes; (**B**,**C**) mRNA expression of senescent cells genes; (**D**) mRNA expression of SASP gene IL10; (**E**) mRNA expression of inflammasome genes. Exact *p* values are indicated between the group bars.

We extracted total RNA from lung tissues of both groups for RT-Q-PCR analysis. Despite using 1 µg total RNA, as with other tissues, the house-keeping gene GAPDH expression level was very low, with CT values between 27 and 32. The IL8 expression was not detectable for any samples. Several other genes had undetectable samples which were not included in the statistical analysis. Fisetin treatment did not significantly change SOD1 and CAT mRNA expression levels (Figure 11A), and showed a down-regulating trend of GLB1 (*p* = 0.169) and P53 expression (*p* = 0.082) along with significantly decreased P21 expression (*p* = 0.028) (Figure 11B,C). Fisetin treatment did not change IL10 expression in the lung tissues (Figure 11D). Fisetin treatment did not significantly change NLRP3, but significantly downregulated TREM2 expression in lung tissues (Figure 11E). Taken together, fisetin treatment significantly decreased P21 and TREM2 expression and showed a decreasing trend of GLB1 and P53 expression in lung tissues.

### 3.10. Effects of Fisetin Treatment on the Gene Expression of the Liver of Old Sheep

**Figure 12:**
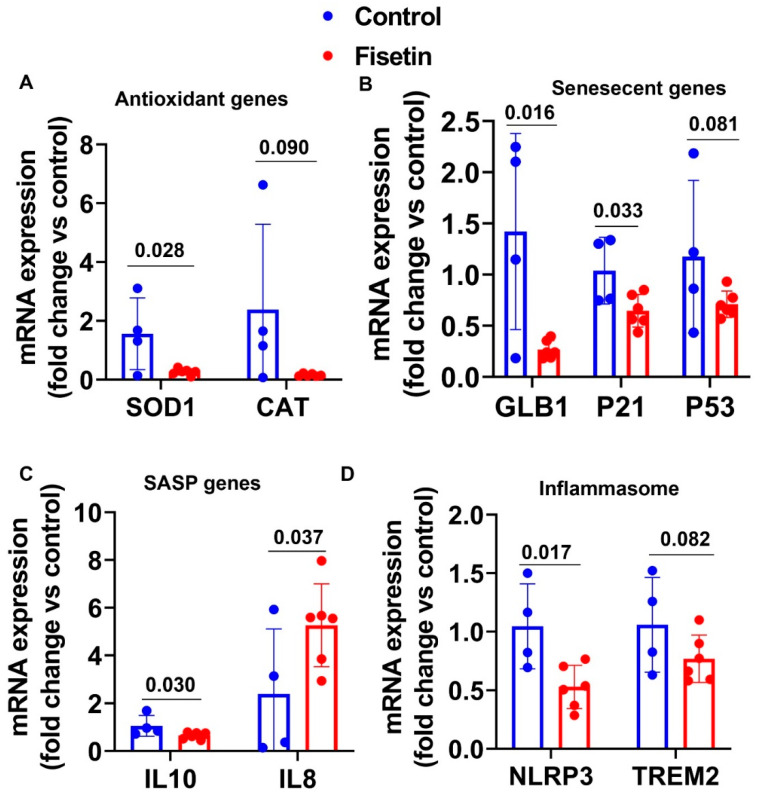
Effects of fisetin treatment on gene expression of the liver: (**A**) mRNA expression of antioxidant genes; (**B**) mRNA expression of senescent genes; (**C**) mRNA expression of SASP gene IL10; (**D**) mRNA expression of inflammasome genes. Exact *p* values are indicated between the groups.

We performed RT-Q-PCR analysis on liver tissues of both groups. Fisetin treatment significantly decreased SOD1 expression (*p* = 0.028) and showed a downregulating trend of CAT gene expression (Figure 12A). Fisetin treatment significantly downregulated mRNA of GLB1 (*p* = 0.016), P21 (*p* = 0.033) and showed a downregulating trend of P53 expression (*p* = 0.082) (Figure 12B). Fisetin treatment decreased IL10 expression (*p* = 0.030) and increased IL8 expression (*p* = 0.037) (Figure 12C). Finally, fisetin treatment significantly decreased the mRNA expression of NRLP3 (*p* = 0.017) and demonstrated a decreasing trend of TREM2 expression (*p* = 0.082) (Figure 12D). Taken together, fisetin treatment significantly decreased SOD1, GLB1, P21, IL10, and NLRP3 while increasing IL8 mRNA expression in the liver.

Taken together, the effects of fisetin treatment on the antioxidant and senescent related genes expressions differ in the various tissues tested, as summarized in Table 3.

### 3.11. Effects of Fisetin Treatment on Plasma MDA and S100B

**Figure 13:**
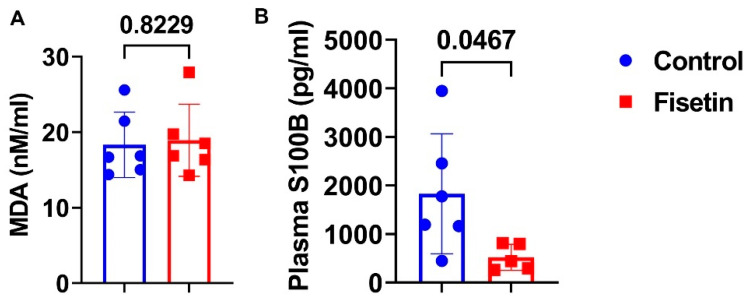
Effects of fisetin treatment on plasma MDA and S100B: (**A**) plasma MDA concentration and (**B**) plasma S100B concentration. Exact *p* values are indicated between the group bars.

We performed plasma analysis for MDA, a product of lipid perioxidation. We did not observe significant difference between the fisetin-treated group and the control group (Figure 13A). However, fisetin treatment significantly decreased S100B expression, a neuronal aging and injury marker (Figure 13B) (*p* = 0.0467).

## 4. Discussion

The most important finding of this study is the discovery of the presence of numerous senescent cells in both the cerebral brain cortex and cerebellum and non-CA area of the hippocampus in old sheep, as demonstrated by SA-β-Gal staining and GL13 staining. The cerebral cortex gray matter had a relative higher number of senescent cells compared to the white matter. Senescent cells were mainly large neurons in both the gray and the white matter of cerebral brain cortex, cerebellum, and non-CA area of the hippocampus. The CA1-4 area of hippocampus has very few cells that become senescent, as demonstrated by GL13 staining and GLB1/NEUN double IF staining. Fisetin treatment at the tested regimen (100 mg/kg, two consecutive days a week for 8 weeks) resulted in a decreasing trend of SA-β-Gal cells in the gray matter of both the cerebral brain cortex and the cerebellum. Fisetin significantly decreased the number of SA-β-Gal cells in the brain cortex white matter compared to control. Furthermore, P16 and GLB1 colocalization with different neuronal markers indicated that fisetin treatment significantly decreased senescent neurons, astrocytes, and microglia in the gray and white matter of the brain cortex and non-CA area of the hippocampus. Fisetin treatment did not affect P16^+^ and GLB1^+^ cells in the NEUN^+^ neurons in the CA1-4 area of the hippocampus due to the presence of very few senescent cells in this area. At the mRNA level, fisetin significantly changes the cellular senescence profile in the liver, with variable effects on cellular senescence in other tissues.

Cellular senescence was recently discovered as a fundamental mechanism of aging and age-related diseases [33,34,35,36]. Targeting senescent cells using senolytic drugs/agents has been shown to improve SASP and frailty and to enhance metabolic function in aged mice [37,38]. Systemic treatment with different senolytic drugs such as the JAK1 inhibitor Ruxolitinib or senolytic cocktail dasatinib plus quercetin (D + Q) for aged mice (20–22 months) prevented age-related bone loss [39]. Senolytic drug treatment improved overall physical function of aged mice and extended the lifespan of old mice by decreasing senescent cells [40]. A human clinical trial showed that D + Q treatment decreased the senescent cell burden in humans, as evidenced by a reduction in adipose tissue senescent cell burden, as determined by a decreases in p16^+^ and p21^+^ cells, SA-β-Gal^+^ cells, and adipocyte progenitors with limited replicative potential [41]. Most previous studies on cellular senescence used rodent animals. However, there is a lack of cellular senescence studies investigating the senescent burden with or without senolytic treatment in the brains of large animals. Our SA-β-Gal staining results indicate that senescent cells are widely present in both the gray matter and white matter of old sheep brains. More senescent cells are present in the gray matter than in white matter. Further, from the morphology of the SA-β-Gal^+^ cells, they are mainly large neurons in these brain regions. GL13 staining in the hippocampus demonstrated the presence of senescent large neurons in the non-CA area but not in neurons in the CA-1-4 areas. These results indicate the presence of senescent cells in various regions of the brains of these old sheep.

Alzheimer’s disease (AD) is the most common form of dementia. No treatments are available to cure, prevent, or delay the progression of AD. Multiple changes associated with brain aging, including neuroinflammation and oxidative stress, contribute to disease development and progression [42]. Many studies have shown the beneficial effects of fisetin on the different neurological disorders via its effects on multiple pathways. These include its anti-inflammatory and antioxidant effects, as well as regulating cell death oxytosis/ferroptosis pathway and the gut microbiome [43,44]. Fisetin has been deemed a promising nutraceutical for treatment of Alzheimer’s disease. A combination of nutraceutical substances and other preventive measures could have significant clinical impact in a multi-layered therapy approach to treat AD [45]. Fisetin treatment has been shown to decrease senescence and SASP in aged or progeria mice as well as to extend lifespan [18]. However, no study has investigated the effects of fisetin on brain cellular senescence in large animal models.

In this study, we have shown that fisetin treatment at the current treatment regimen decreased SA-β-Gal-positive cells in the gray matter and white matter of the cerebral brain cortex and in the gray matter in the cerebellum. In addition, fisetin treatment significantly decreased GL13^+^ neurons in the non-CA area of hippocampus. However, fisetin treatment did not eliminate all senescent cells in any of these regions of the brain, and many SA-β-Gal-positive cells were still present in the fisetin-treated sheep brain regions. To determine which cells become senescent in the brain, we performed colocalization of P16 and GLB1 with the neuronal marker NEUN, astrocyte marker GFAP, and microglia marker IBA1. We found that fisetin treatment significantly decreased P16^+^NEUN^+^/Total NEUN^+^, P16^+^GFAP^+^/Total GFAP^+^, and P16^+^IBA1^+^/Total IBA1^+^ cells percentages in the cerebral brain cortex in both gray and white matter. Consistent with P16 double IF staining, GLB1 double IF staining with different neuronal markers revealed that fisetin treatment significantly decreased senescent neurons, astrocytes, and microglia in the gray and white matter of the brain cortex.

In the hippocampus area of the brain, our P16 and GLB1 double IF staining revealed that fisetin treatment significantly decreased senescent microglia (P16^+^/IBA1^+^/Total IBA1^+^ cells percentage and GLB1^+^IBA1^+^/total IBA1^+^ cells percentage) in the non-CA area of the hippocampus. Fisetin treatment significantly decreased senescent astrocytes in the non-CA area of the hippocampus, as demonstrated by the significant decrease in GLB1^+^GFAP^+^/total GFAP^+^ cells percentage, with a decreasing trend of P16^+^GFAP^+^/Total GFAP^+^ cells percentage of astrocytes in the non-CA-area of the hippocampus. The insignificant (*p* = 0.1076) decrease of P16^+^GFAP^+^/total GFAP^+^ cells percentage by fisetin treatment is likely related to the differences between P16 and GLB1 in labeling senescent cells. It is recognized that not all P16^+^ cells are senescent cells, although most senescent cells express P16 [32]. Interestingly, fisetin treatment did not affect senescent cells in the CA1-4 areas, as revealed by GL13 staining, P16^+^NEUN^+^/total NEUN^+^ cells percentage, and GLB1^+^NEUN^+^/total NEUN^+^ cell percentage. This is most likely due to the fact that very few cells (less than 2%) become senescent in the CA1-4 area, as revealed by GL13 staining and GLB1/NEUN double IF staining (Figure 1I,J and Figure 6A,B). However, approximately 80% of CA 1-4 area neurons were P16^+^. Therefore, P16 is a less reliable senescent marker, but its expression is necessary to maintain cells in a quiescent state. Whether other senolytic agents/drugs are more effective than fisetin in our animal model will require further study. Indeed, a recent clinical trial data using D + Q treatment to prevent Alzheimer’s disease showed promising results in decreasing senescence-related cytokines and chemokines [46].

Furthermore, it is interesting that we found quite a high number of SA-β-Gal^+^ neurons in the brain cortex. It is likely that not all SA-β-Gal^+^ cells are senescent, because previous study has demonstrated many SA-β-Gal^+^ are negative for markers of double-strand DNA breaks (DSBs) [47]. Regardless, the specificity of SA-β-Gal^+^ staining in neurons, together with other senescent markers used in this study, indicate that fisetin is effective in reducing senescent cells in brain tissues.

In addition, we found that fisetin treatment significantly decreased S100B protein in the plasma. S100B has been well accepted as a brain aging and neural injury marker. However, mounting evidence indicates that S100B serves as a Damage-Associated Molecular Pattern (DAMP) molecule, which at high concentrations can trigger tissue damage [48]. In many neurological disorders, S100B levels correlate with clinical or toxic parameters; thus, S100B can serve as a therapeutic target for neurological diseases [48,49]. These results further indicate the beneficial effects of fisetin for brain aging.

It is worthy to emphasize that fisetin decreased P16^+^IBA1^+^/Total IBA1^+^ cells percentage in microglia in both the cerebral brain cortex and the hippocampus, although we used different P16 antibodies. This result was further validated by GLB1/IBA1 double staining, which showed similar results. Microglia are from a macrophage lineage in the brain [50]. It is known that both activated inflammatory microglia and senescent microglia secrete TNFα, IL1β, and IL6 [51]. Fisetin treatment inhibited the upregulation of IL1β induced by LPS/IFN-γ- or peptidoglycan-induced inflammatory mediator in microglia cells [52]. Fisetin induced endogenous anti-oxidative enzyme HO (heme oxygenase)-1 expression through the PI-3 kinase/AKT and p38 signaling pathways in microglia [52]. Further, fisetin significantly attenuated inflammation-related microglial activation and coordination deficit in mice in vivo [52]. Fisetin treatment has been reported to alleviate intracerebral hemorrhage (ICH)-induced brain injury by downregulating proinflammatory cytokines and attenuating NF-κB signaling and preventing microglia activation [53].

We found that fisetin treatment affected the antioxidant genes SOD1 and CAT differently in different organs. We found that fisetin treatment decreased SOD1 expression in the liver and spleen, and increased CAT expression in the spleen but did not significantly change these two gene expressions in the other tissues tested. Further, fisetin treatment at the current regimen did not affect the plasma MDA level. Fisetin is well known for its antioxidant effects by increasing glutathione (GSH), mainly through activating antioxidant transcription factor NRF2 [4,6]. In a vascular dementia model, fisetin treatment attenuated histological injury, MDA levels, inflammasome pathway activation, apoptosis, and increased brain derived neural growth factor (BDNF) expression, reduced astrocyte and microglial activation, and cognitive deficits [54]. However, it has been reported that fisetin’s neuroprotective and antioxidant effects can be negatively affected by metal such as iron and copper. Fisetin could only reduce cell death induced by iron and copper in response to treatments that lower GSH levels (in the presence of glutamate). Fisetin treatment is less effective when the metal toxicity is combined with other inducers of oxidative stress, such as hydrogen peroxide (H_2_O_2_) or t-butyl peroxide. This is because the effects of fisetin on induction of the antioxidant transcription factor Nrf2 can be blocked by iron, but not by copper [55]. A previous study using pressure overload induced cardiac hypertrophy model showed that fisetin markedly reduced ROS by increasing expression of SOD1, CAT and HO1 [56]. However, our results showed that fisetin treatment increased CAT expression in the spleen (*p* = 0.053), and decreased SOD1 expression in the liver and spleen likely due to we used different treatment regimen.

Finally, we found variable effects of fisetin on the expression of GLB1 in different organs. We found that fisetin treatment significantly decreased GLB1 in the liver, and only found a decreasing trend of GLB1 expression in the spleen, lung, and heart. We found different changes of inflammasome gene expressions after fisetin treatment, with the liver and lung having the most significantt decrease in NLRP3 and TREM2 expression.

This is likely due to the fact that IV infusion of fisetin allows fisetin to enter the liver and lung more readily than other organs. Furthermore, we used a “hit-and run” strategy specific to senolytic treatment instead of regular drug treatment [18,41]. This treatment regimen does not produce sustained serum or tissue levels of fisetin when compared to the daily fisetin administration. Fisetin has been shown to downregulate the activation of the NLRP3 inflammasome induced by LPS and ATP (LPS/ATP) and the subsequent maturation of IL-1β [57]. Fisetin activated mitophagy and prevented the accumulation of damaged mitochondria and the excessive production of mitochondrial ROS [57]. Treatment of LPS/ATP-stimulated zebrafish with fisetin facilitated recovery of the impaired heart rate, decreased the recruitment of macrophages to the brain, and gradually downregulated the expression of inflammasome-related genes in a p62-dependent manner [57]. Our results show that fisetin significantly decreased NLRP3 expression in the liver and TREM2 expression in the lung in aged sheep, without causing any ROS challenge.

## 5. Conclusions

In summary, our results indicate that senescent cells are widely present in the gray and white matter of the cerebral brain cortex and cerebellum as well as in the non-CA area of the hippocampus in old sheep. Very few senescent cells are present in the CA1-4 areas of the hippocampus. Fisetin treatment decreased senescent cells in the gray matter of the brain cortex and cerebellum as well as large neurons (GL13 staining) in the non-CA area of the hippocampus. Further, fisetin treatment significantly decreased P16^+^ and GLB1^+^ neurons, astrocytes, and microglia cells in both gray and white matter of the cerebral brain cortex, and reduced GLB1^+^ microglia, astrocytes, and GLB1^+^ neurons in the non-CA area of the hippocampus. Fisetin treatment significantly decreased plasma S100B. At the mRNA level, fisetin treatment downregulated GLB1 expression in the liver, with a decreasing trend of GLB1 expression in the lung, heart, and spleen tissues. Fisetin treatment significantly decreased TREM2 expression in the lung, with a decreasing trend of TREM2 expression in the brain, liver, and spleen. Fisetin treatment significantly decreased NLRP3 expression in the liver. Surprisingly fisetin treatment significantly decreased SOD1 expression in the liver and spleen, with a decreasing trend of SOD 1 expression in the bone marrow. Fisetin treatment upregulated CAT expression in the spleen. These results suggest that fisetin treatment represents a promising therapeutic strategy for age-related diseases.

## Figures and Tables

**Table 1:** Primer information.

Accession #	Gene ID	Forward Primer (5′-3′)	Reverse Primer (5′-3′)	Product Size (bp)
XM_018065254.1	Goat GFAP	caggatctgctcaacgtcaa	atctccacggtcttcaccac	197
XM_015102219.3	Sheep GLB1	agtccccctacctacgcact	ggtcgaagtcaccaggatgt	213
NM_001145185.2	Sheep SOD1	ggttccacgtccatcagttt	tttgtcagccttcacattgc	143
XM_005690077.3	Goat CAT	ctcgtccaggatgtggtttt	gagcctgattctccagcaac	215
XM_042234806.1	Sheep NEFH	acatcgcatcctaccaggag	cagcgatctcaatgtccaga	135
XM_015093090.3	Sheep NEFL	aagcgcatagacagcctgat	ctctcggtcagcacagtgaa	242
HM043737.1	Sheep GAPDH	acagtcaaggcagagaacgg	ggttcacgcccatcacaaac	235
X78306.1	Sheep IL8	tcgatgccaatgcataaaaa	ttggggtctaagcacacctc	147
NM_001009327.1	Sheep IL10	tgttgacccagtctctgctg	ttcacgtgctccttgatgtc	136
FJ943992.1	Sheep P21	gagagcgatggaacttcgac	agtggtcctcctgagacgtg	186
FJ855223.1	Sheep P53	cctgctcccgtactcagaag	ctggcagaacagcttgttga	247
XM_042250404.1	Sheep NLRP3	ctgtgcacacggtggtattc	ctctgagtcccaaggctcac	157
XM_004018807.5	Sheep TREM2	agcctttcggaagaggagag	agctggtaacctgggttgtg	179

**Table 2:** Summarized results of the effect of fisetin on cellular senescence of the different brain regions (*p* values).

Staining Methods	Brain Cortex Gray Matter (*p* Value)	Brain Cortex White Matter (*p* Value)	Hippocampus CA1-4 Area (*p* Value)	Hippocampus (Non-CA Area) (*p* Value)	Cerebellum Gray Matter (*p* Value)	Cerebellum White Matter (*p* Value)
SA-β-Gal staining	0.1449	**0.0306**	N/D	N/D	0.1804	0.9891
GL13 staining	N/D	N/D	0.1685	**0.0022**	N/D	N/D
P16^+^/NEUN^+^ (%)	**0.0079**	N/A	0.7446	N/D	N/D	N/D
P16^+^/NEUN^−^/DAPI (%)	N/A	**0.0002**	N/D	N/D	N/D	N/D
P16^+^/GFAP^+^ (%)	**0.0255**	**0.0261**	N/A	**0.1076**	N/D	N/D
P16^+^/IBA1^+^ (%)	**0.0407**	**0.0004**	N/A	**0.0102**	N/D	N/D
GLB1^+^/NEUN^+^ (%)	**0.0048**	N/A	0.4407	**0.0332**	N/D	N/D
GLB1^+^/GFAP^+^ (%)	**0.0375**	**0.0008**	N/A	**0.0429**	N/D	N/D
GLB1^+^/IBA1^+^ (%)	**0.0029**	**<0.0001**	N/A	**0.0025**	N/D	N/D

Green font indicates decrease in quantification. Bold green font indicates significant decrease in quantification. Non-bold green font *p* values indicate only a decreasing trend in quantification. N/A, not applicable. N/D, not determined.

**Table 3:** Summarized RT-Q-PCR results of different organs after fisetin treatment (*p* values).

Genes Name	Brain Cortex (*p* Value)	Heart (*p* Value)	Spleen (*p* Value)	Bone Marrow (*p* Value)	Lung (*p* Value)	Liver (*p* Value)
SOD1	NS	NS	**0.017**	0.105	NS	**0.028**
CAT	NS	NS	0.053	NS	NS	0.090
GLB1	NS	0.192	0.083	NS	0.169	**0.016**
P21	0.127	NS	NS	NS	**0.028**	**0.033**
P53	NS	NS	NS	**0.046**	0.081	0.081
IL10	NS	0.070	NS	NS	NS	**0.030**
IL8	NS	0.069	NS	NS	N/A	**0.037**
NLRP3	NS	0.121	NS	NS	NS	**0.017**
TREM2	0.136	NS	0.098	0.073	**0.029**	0.082

The green color indicates downregulation. The red color indicates upregulation. Bold font *p* values indicate significant differences. Non-bold font *p* values indicate only difference trends. NS indicates no significant differences. N/A means not applicable.

## Data Availability

Original data will be available from corresponding authors upon request.
